# Prevalence and factors associated with depression among hospital admitted patients in South Ethiopia: cross sectional study

**DOI:** 10.1186/s13104-019-4109-3

**Published:** 2019-02-04

**Authors:** Bereket Duko, Marta Erdado, Jemal Ebrahim

**Affiliations:** Faculty of Health Sciences, College of Medicine and Health Sciences, Hawassa University, Hawassa, Ethiopia

**Keywords:** Prevalence, Depression, Associated factor, Inpatient, Ethiopia

## Abstract

**Objective:**

Identifying factors associated with depression will help health care providers to design programs which improve quality of services provided to the inpatients. The aim of this study was to assess the prevalence and factors associated with depression among admitted patients at Adare General Hospital, Hawassa, Ethiopia, 2017/2018.

**Result:**

The mean age of the respondents was 38 years (SD ±15.39). The prevalence of depression was 38%. After adjusting for possible confounding variables; age category of 18–24 years [AOR = 1.24, 95 CI (1.08–5.73), having cardiovascular disease [AOR = 2.20), 95 CI (1.38–7.28)] and being in surgical ward [AOR = 1.92), 95 CI (2.13–4.12)] had statistically significant association with depression.

## Introduction

Depression is an emotional state which is experienced by most people sometime in life and can manifest as the blues or sadness, grief, mourning, no pathologic or neurotic or in the form of hyperactivity and manic behavior [1, 2]. The estimated of prevalence of depressive disorder is ranges 5–10% in care giving settings and it ranks the fourth leading causes of disability in world [3, 4].

Studies revealed that family history of depression, substance abuse, suicide, impulsive behavior, severe or chronic medical conditions, being female sex, intimate partner violence and sexual abuse are associated factors of depression [5, 6].

According to world health organization estimate, averagely 15% of global populations experience depression once or more in their stay of life [7, 8]. Depression could result significant morbidity and mortality to hospital admitted patients which is expected to be the second leading causes of disability-adjusted life years [9]**.**

Depressive disorders are more common in hospital admitted patients. One study in Kenya revealed that 42% of inpatients experienced mild to severe depression [10]. In addition to this, other studies which are conducted in different setting on the prevalence of depression among admitted patients also showed that 48.5% in Pakistan [11], 29.4% in Addis Ababa, Ethiopia [12] and 54.6% in Mekele, Ethiopia [13]. Being female sex, age > 60 years, being divorced, being in age 35–44, being in surgical ward and medical ward are factors have statically significant association with depression [10–13]. Therefore, this study aimed to assess the prevalence and associated factors of depression in hospital admitted patient.

## Main text

### Study setting and population

Hospital based cross sectional study design was employed from May 2017 to June 2017 at Adare General Hospital, Hawassa, Ethiopia. Adare general hospital is one governmental hospitals in Hawassa, which is located 273 km away from Addis Ababa, the capital city of Ethiopia. A total 203 admitted patients who are ≥ 18 years were included for the study from those who unable to communicate because of the severity of illness and with diagnosis of major depressive disorders and other mental illness, and those who were in the operation room during the study period were excluded from the study. We had used stratified sampling technique to identify the specific ward of patient admission. Systematic sampling technique was used to recruit patients from each strata.

### Data collection

We have used semi-structured questionnaire to collect socio-demographic, substance use, clinical factors and use Beck depression inventory scale to assess depression. Data was collected through face to face interview. Beck depression inventory scale was used as a screening tool for depression in many epidemiological studies across the world. It consists of 21 questions with a likert scale of 0–3 providing a total score of 63. According to the revised version of Beck-II in 1996, the score from 0–13 is considered as normal, 14–19 as mild, 20–28 as moderate and 29–63 as severe depression. Those patients who scored moderate and severe considered to have depression [14].

### Data processing and analyses

The collected data was edited, coded, entered into EPI info version 7.1 and was analyzed using SPSS version 20. We had conducted binary logistic regression to identify risk factors of depression.

### Results

#### Socio-demographic characteristics of the study participants

Among 203 study participants, 194 patients were involved in the study with mean age of 38 years (SD ± 15.39) and resulted a response rate of 95.6%. Out of 194 respondents, 105 (54.1%) were male and 96 (49.5%) were single (Table 1**).**

**Table 1:** Distribution of respondents by their socio-demographic characteristic, Adare General Hospital, Hawassa, Ethiopia, 2017

Category	Frequency (n = 194)	Percent (%)
Age		
18–24	38	19.6
25–34	59	30.4
35–44	40	20.6
45–54	24	12.4
≥ 55	33	17.0
Sex		
Male	105	54.1
Female	89	45.9
Marital status		
Single	65	33.5
Married	96	49.5
Divorced	14	7.2
Widowed	19	9.8
Ethnicity		
Sidama	65	33.5
Wolaita	53	27.3
Oromo	31	16.0
Amhara	24	12.4
Others	20	10.3
Religion		
Orthodox	67	34.5
Muslim	89	45.9
Protestant	25	12.9
Catholic	13	6.7
Education		
Unable to read and write	24	12.4
No formal education	15	7.7
Primary	48	24.7
Secondary	36	18.6
College and above	70	36.1
Occupation		
Governmental employee	34	17.5
House wife	21	10.8
Farmer	27	13.9
Non-governmental	42	21.6
Merchant	36	18.6
Student	28	14.4
Others	40	20.6

#### Clinical and substance use related characteristics of the study participants

From the total study participants, 31 (16%) were currently smoking cigarettes, 48 (24.7%) were drinking alcohol and 44 (22.7%) were chewing khat (chat) (Table 2).

**Table 2:** Clinical and substance related factors among admitted patients, Adare General Hospital, Hawassa, Ethiopia, 2017

Variables	Frequency	Percent
Illness		
Cardiovascular disorder		
Yes	38	19.6
No	156	80.4
Neurologic disorder		
Yes	5	2.6
No	189	97.4
Gastro-intestinal disorder		
Yes	52	26.8
No	142	73.2
Substance use		
Consuming tobacco products		
Yes	31	15.9
No	163	84.1
Drinking alcohol		
Yes	48	24.7
No	146	75.3
Chewing chat		
Yes	44	22.7
No	150	77.3
Ward		
Surgical	58	29.9
Gynaecology	54	27.8
Medical	82	42.3

#### Prevalence and factors associated depressive symptom among the study participants

Using Beck depression inventory scale with cut-off point ≥ 21 revealed that the prevalence of depression was 38%. Being in age category of 18–24, having cardiovascular disease and being admitted in surgical ward were associated with depressive symptom according to binary logistic regression analysis (Table 3).

**Table 3:** Factors associated with depressive symptom among admitted patients at Adare General Hospital, Hawassa, Ethiopia, 2017

Variables	Depression		COR (CI)	AOR (CI)
	Yes	No		
Age category				
18–24	28	10	1.60 (1.07–9.56)	1.24 (1.08–5.73)
25–34	18	41	0.25 (0.10–4.62)	0.34 (0.12–2.43)
35–44	14	26	0.30 (0.19–6.80)	0.38 (0.13–1.09)
45–54	11	13	0.48 (0.16–1.14)	0.44 (0.14–1.41)
≥ 55	21	12	1	1
Cardiovascular disease				
Yes	24	14	0.275 (0.13–0.57)	2.20 (1.38–7.28)
No	50	106	1	1
Gastrointestinal disease				
Yes	26	26	0.51 (0.26–0.97)	0.44 (0.27–5.47)
No	94	48	1	1
Ward				
Surgical	38	20	3.87 (1.26–5.35)	1.92 (2.13–4.12)
Gynaecology	18	36	1.01 (0.85–2.87)	1.01 (0.85–2.87)
Medical	27	55	1	1

### Discussion

The findings of this study revealed that the overall prevalence of depression among hospital admitted patients was 38%. The study finding was lower than study conducted among hospital admitted patients in Ethiopia [13], in Spain [15], and in Western Asia [16]. On the other hand, the current finding is higher than the study conducted in Paris metropolitan area [17] and in Sweden [18]. The Difference might be difference in sample size, data collection tool and socio-cultural difference.

Being in age category of 18–24 had significant association with depressive symptom. This finding is congruent with other studies [13]. People aged 18–24 years have the highest prevalence of mental disorders of any age group. This is supported by many more literatures worldwide. There was no statistically significant association between depression and gender in this study.

Patients who had cardiovascular disorder were 2.2 times more likely to have depressive symptom when compared to the patients who had no cardiac problems. This is due to the fact that depression is more common in cardiac patients, this would seem that either depression leads to cardiovascular disorder or cardiovascular disorder leads to depression or may be both [19, 20].

Patients who were admitted in surgical ward for surgical management were 3.87 times more likely to have depression when compared to patient admitted to medical ward. This might be due to the fact that having surgical management by itself creates stress, admission in hospital is stressful and some other different factors like a kind of disease, hospital environment, patient’s concern about being away from their family and missing their job.

### Conclusion

According to the current study finding, depression was high in admitted patients in the study setting. Being age category 18–24, having cardiovascular disorder and being in surgical ward had statistically significant association with depression. They recommends to have further research on probable risk factors of depression to strengthen the current result.

## Limitation of the study

We didn’t do detailed validation study for Beck’s depression inventory scale. This might under or overestimate the study findings. The study didn’t include detailed clinical factors which might contribute for depressive symptom.
